# A Carbohydrate Moiety of Secreted Stage-Specific Glycoprotein 4 Participates in Host Cell Invasion by *Trypanosoma cruzi* Extracellular Amastigotes

**DOI:** 10.3389/fmicb.2018.00693

**Published:** 2018-04-10

**Authors:** Pilar T. V. Florentino, Fernando Real, Cristina M. Orikaza, Julia P. C. da Cunha, Francisca N. L. Vitorino, Esteban M. Cordero, Tiago J. P. Sobreira, Renato A. Mortara

**Affiliations:** ^1^Department of Microbiology, Instituto de Ciências Biomédicas, Universidade de São Paulo, São Paulo, Brazil; ^2^Institut Cochin, INSERM U1016, Paris, France; ^3^Department of Microbiology, Immunology and Parasitology, Escola Paulista de Medicina, Universidade Federal de São Paulo, São Paulo, Brazil; ^4^Special Laboratory of Cell Cycle, Center of Toxins, Immune-Response and Cell Signaling (CeTICS), Butantan Institute, São Paulo, Brazil; ^5^Facultad de Ciencias, Centro de Genómica y Bioinformática, Universidad Mayor, Santiago, Chile; ^6^Bindley Bioscience Center, Purdue University, West Lafayette, IN, United States

**Keywords:** *Trypanosoma cruzi*, extracellular amastigotes, Ssp-4 glycoprotein, galectin-3

## Abstract

*Trypanosoma cruzi* is the etiologic agent of Chagas’ disease. It is known that amastigotes derived from trypomastigotes in the extracellular milieu are infective *in vitro* and *in vivo*. Extracellular amastigotes (EAs) have a stage-specific surface antigen called Ssp-4, a GPI-anchored glycoprotein that is secreted by the parasites. By immunoprecipitation with the Ssp-4-specific monoclonal antibodies (mAb) 2C2 and 1D9, we isolated the glycoprotein from EAs. By mass spectrometry, we identified the core protein of Ssp-4 and evaluated mRNA expression and the presence of Ssp-4 carbohydrate epitopes recognized by mAb1D9. We demonstrated that the carbohydrate epitope recognized by mAb1D9 could promote host cell invasion by EAs. Although infectious EAs express lower amounts of Ssp-4 compared with less-infectious EAs (at the mRNA and protein levels), it is the glycosylation of Ssp-4 (identified by mAb1D9 staining only in infectious strains and recognized by galectin-3 on host cells) that is the determinant of EA invasion of host cells. Furthermore, Ssp-4 is secreted by EAs, either free or associated with parasite vesicles, and can participate in host-cell interactions. The results presented here describe the possible role of a carbohydrate moiety of *T. cruzi* surface glycoproteins in host cell invasion by EA forms, highlighting the potential of these moieties as therapeutic and vaccine targets for the treatment of Chagas’ disease.

## Introduction

The flagellated protozoan *Trypanosoma cruzi* is the etiologic agent of Chagas’ disease and is responsible for an estimated 6–7 million individuals infected worldwide, mostly in Latin America (World Health Organization [WHO], 2017). This parasite has four defined morphological stages: two infective forms called metacyclic and bloodstream trypomastigotes and two replicative forms known as amastigotes and epimastigotes (Clayton, 2010). Although *T. cruzi* amastigotes are usually found in the cytoplasm of infected cells of the mammalian host, these forms can also be found in the extracellular milieu due to trypomastigote differentiation or early lysis of infected cells (Andrews et al., 1987; Ley et al., 1988) or due to cytolysis at inflamed sites of infection during the chronic stage of Chagas’ disease (Scharfstein and Morrot, 1999).

These extracellular amastigotes (EAs) are proxies for their intracellular counterparts as they share morphological and immunochemical markers and are capable of invading and sustaining infection cycles in mammalian cells (Nogueira and Cohn, 1976; Ley et al., 1988). However, unlike the infective trypomastigote forms, EAs invade HeLa cells in an actin-dependent mechanism, forming a phagocytic cup that surrounds these parasites (Mortara, 1991; Procópio et al., 1999), suggesting that EAs display functionally distinct membrane proteins that interact with a different set of host cell receptors. The membrane proteins on the surfaces of EAs are recognized by host cell receptors, and the roles of these proteins in actin-dependent invasion remain elusive.

Kahn et al. (1996) have observed that amastigotes, but not trypomastigotes or epimastigotes, interact with host macrophages via mannose surface receptors (MRIs). The cell surface protein galectin-3 (Gal-3), which belongs to the galectin family and recognizes β-galactosides, has been previously implicated in the interaction of *T. cruzi* with host cell membranes (Moody et al., 2000; Kleshchenko et al., 2004; Vray et al., 2004; Pineda et al., 2015). In addition, Machado et al. (2014) observed the recruitment of galectin-3 at invasion sites of EAs in macrophage cells.

The EAs from group I strains (such as the G strain) were found to enter mammalian cells much more efficiently than parasites from groups II (Y strain) or VI (CL strain) (Fernandes and Mortara, 2004; Mortara et al., 2005; da Silva et al., 2006; Fernandes et al., 2007). Different studies have shown that the expression of protein and carbohydrate epitopes varies between *T. cruzi* strains and that these variations are correlated with parasite infectivity (Mortara et al., 1988, 1992; Verbisck et al., 1998; da Silva et al., 2006; Yoshida, 2006). We have previously observed two secreted proteins from EAs, p21 and mevalonate kinase, that mediate host cell signaling during invasion (da Silva et al., 2009; Ferreira et al., 2016). EAs also express on their surfaces a major glycoprotein, stage-specific protein 4 (Ssp-4), initially described by Andrews et al. (1987). This protein is gradually released into extracellular milieu, which is mediated by an endogenous phosphatidylinositol-specific phospholipase C (PI-PLC) (Andrews et al., 1988). We have demonstrated that Ssp-4 was also released in membrane trails when EAs were attached to poly-L-lysine-coated glass (Barros et al., 1996). Two monoclonal antibodies detect different epitopes of Ssp-4, mAb2C2 (Andrews et al., 1987) and mAb1D9, this last one recognizes a carbohydrate epitope (Barros et al., 1997). Moreover, highly infective EAs from the G strain showed reduced parasite invasion when incubated with mAb1D9 (da Silva et al., 2006), suggesting the involvement of Ssp-4 in host cell invasion.

Although limited in their ability to mimic intracellular amastigotes, EAs present the same set of stage-specific surface antigens as Ssp-4 (Andrews et al., 1987), the structural and functional features of which are not yet fully understood. In the present report, we have revealed the Ssp-4 protein sequence and observed a correlation between the expression of protein and carbohydrate epitopes and the strain infectivity profile. Our findings suggest that parasite infectivity correlates with posttranslational modifications in Ssp-4, resulting in the expression of distinct carbohydrate epitopes on EA surfaces. We also demonstrated that the highly infective G strain secretes an Ssp-4 epitope (recognized by mAb1D9) into vesicle trails adhered to HeLa cells, which may contribute to galectin-3 recruitment during host cell invasion by EAs.

## Results

### Ssp-4 Glycosylation Is Associated With Host Cell Invasion by *T. cruzi* EAs

**FIGURE 1:**
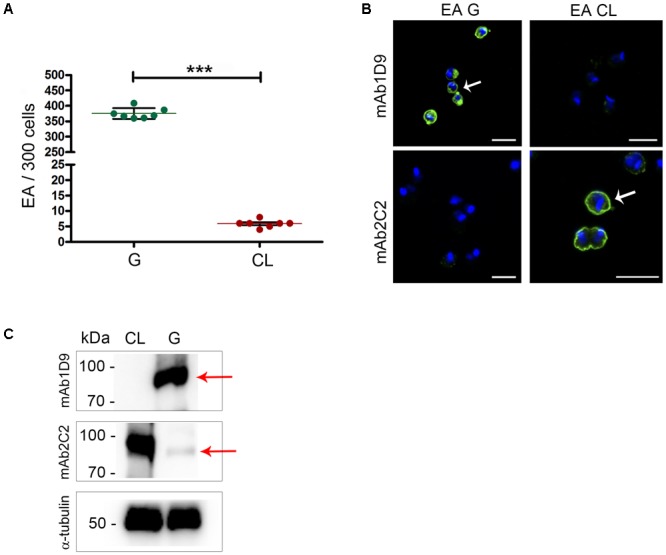
Ssp-4 epitope expression correlates with infectivity of extracellular amastigotes from the G and CL strains. **(A)** Number of internalized parasites in 300 cells per coverslip. Extracellular amastigotes (EAs) from the G and CL strains were incubated with HeLa cells (MOI 10:1) for 2 h. Mean and SD are represented in the graphic. Three independent experiments were performed in duplicate. ^∗∗∗^*p* < 0.001. **(B)** EAs of the G or CL strains immunostained with mAb1D9 and mAb2C2 (green); nuclei and kinetoplasts were labeled with DAPI (blue). White arrows indicate the distribution of Ssp-4 epitopes on parasite cell surfaces. Scale bar: 5 μm. **(C)** Western blotting of EAs from the G and CL lysates was incubated with mAb2C2 and mAb1D9. Red arrows indicate the Ssp-4 band. As a control, EA lysates were incubated with anti-α-tubulin antibody.

Host cell invasion is a property exhibited by EAs of some by *T. cruzi* strains maintained *in vitro*. First, we confirmed that the parasite strains G and CL can be categorized as infectious and less-infectious EA strains, respectively, based on HeLa cell invasion assays. **Figure 1A** shows that the number of internalized parasites from the G strain within HeLa cells is two logs higher (*p* < 0.001) than the number from the CL strain, allowing us to classify these strains as infectious and less infectious, respectively.

Next, the expression of Ssp-4 on the surfaces of infectious and less infectious EAs was assessed by immunofluorescence using two monoclonal antibodies specific for the Ssp-4 antigen, namely, mAb2C2 and mAb1D9, the latter being employed to identify carbohydrate epitopes associated with Ssp-4 (Barros et al., 1997). The carbohydrate epitope recognized by mAb1D9 is highly expressed in infectious EAs and is absent in less infectious EAs; conversely, mAb2C2 only recognizes Ssp-4 on the surfaces of less infectious strains (**Figure 1B**). This result was reproduced in EA lysates immunostained with mAb1D9 and mAb2C2 by Western blotting (**Figure 1C**): while the carbohydrate epitope recognized by mAb1D9 is completely absent in the less-infectious CL strain, the epitope recognized by mAb2C2 is detected in both strains, although this recognition is weak in EAs of the infectious G strain (**Figure 1C**, arrow).

**FIGURE 2:**
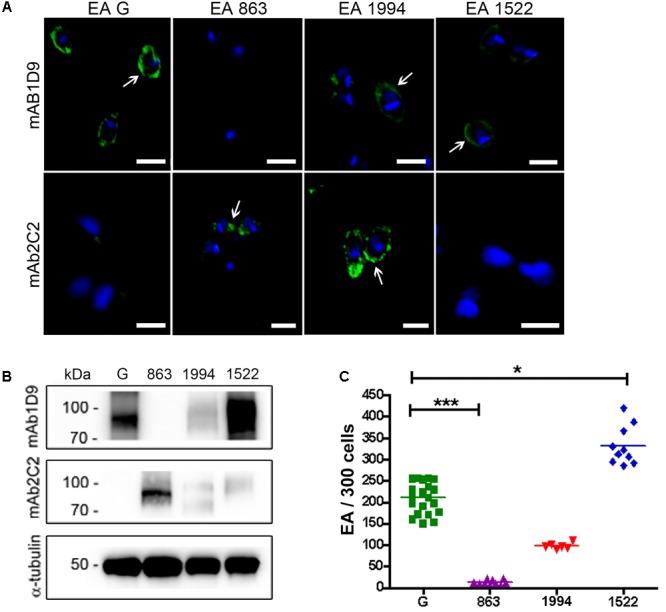
Ssp-4 epitope expression correlates with infectivity of *T. cruzi* isolates. **(A)** Immunofluorescence of extracellular amastigotes (EAs) from the G strain and the Tc863 (863), Tc1994 (1994), and Tc1522 (1522) isolates with mAb1D9 or mAb2C2 (green); nuclei and kinetoplasts were DAPI labeled (blue). White arrows indicate the distribution of carbohydrate epitopes on parasite cell surfaces. Scale bars: 3 μm. **(B)** Western blotting of *T. cruzi* EAs from the G strain and the Tc863, Tc1994, and Tc1522 isolates (863, 1994, and 1522, respectively) incubated with mAb2C2 and mAb1D9; anti-α-tubulin antibody was used for normalization. **(C)** Number of internalized parasites (EAs) in 300 cells per coverslip of *T. cruzi* isolates. Three independent experiments were performed in triplicate or duplicate. ^∗^*p* < 0.05; ^∗∗∗^*p* < 0.001.

The inverse correlation between the detection of the Ssp-4 carbohydrate epitope by mAb1D9 and the infectivity of EAs was investigated using other *T. cruzi* strains. **Figures 2A,B** show the membrane distribution and expression of Ssp-4 epitopes (detected by mAb2C2 and mAb1D9 by immunofluorescence and Western blotting, respectively) in *T cruzi* strains Tc863 (Tc III), Tc1522 (Tc I), and Tc1994 (TcBat) compared with the infectious G strain (Tc I). Highly infective EAs from the G and Tc1522 strains presented higher reactivity to mAb1D9 and lower reactivity to mAb2C2 (**Figures 2B,C**). Conversely, Tc863 exhibited low infectivity and lacked the carbohydrate epitope recognized by mAb1D9; however, this strain exhibited strong reactivity to mAb2C2. Tc1994 (TcBat) showed intermediate infectivity compared with the other isolates and expressed epitopes recognized with similar strength by both mAb1D9 and mAb2C2.

### Ssp-4 Expression Does Not Correlate With EA Infectivity

**FIGURE 3:**
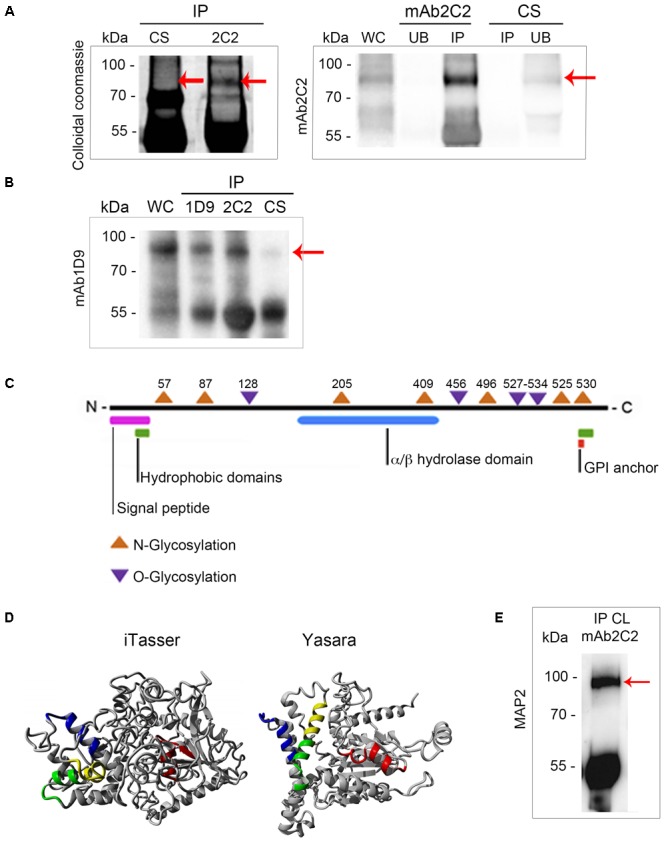
Ssp-4 identification by mass spectrometry revealed a hypothetical protein from the CL Brener database that was more highly expressed in the CL strain. **(A)** Immunoprecipitation (IP) with mAb2C2 or control serum (CS) in protein extracts of extracellular amastigotes (EAs) from the CL strain was performed in the presence of protein A-Sepharose. Colloidal Coomassie-stained polyacrylamide gel of the IP with mAb2C2 or CS and Western blotting with mAb2C2 of the whole cell lysate (WC) from extracellular amastigotes (EAs) of the CL and unbound (UB) fraction and IP of 2C2 and CS. Red arrows indicate the band of 93 kDa. **(B)** IP performed with mAb1D9, mAb2C2, and CS in protein extracts of EAs from the G strain. Western blotting of the WC from the G strain and IPs with mAb1D9. Red arrow indicates the band of 93 kDa. **(C)** Scheme of the hypothetical protein bearing the Ssp-4 epitope identified by mass spectrometry, revealing domains encountered by *in silico* analysis. **(D)** Structural models of the Ssp-4 putative protein predicted by I-Tasser and Yasara. MAP2 peptides are in yellow. In green, blue, and red are the other peptides selected by I-Tasser and Yasara model (MAP1, MAP3, and MAP4, respectively). **(E)** IP of protein extracts of EAs of CL with mAb2C2, subsequently probed with MAP2 in Western blotting.

As we have established that the presence of the Ssp-4 carbohydrate epitope recognized by mAb1D9 correlates with EA infectivity, we sought to identify the Ssp-4 sequence and evaluate whether the mRNA and protein expression of Ssp-4 is also correlated with host cell invasion. We employed an immunoprecipitation assay on lysates of less-infectious EAs by using either mAb2C2 or control serum and specifically detected a band of 93 kDa associated with Ssp-4 (**Figure 3A**). Western blotting with mAb2C2 confirmed the presence of a band corresponding to a 93-kDa protein in the mAb2C2 immunoprecipitates that is absent when immunoprecipitation is performed using control serum (red arrows in **Figure 3A**). Immunoprecipitation of G strain lysates using mAb1D9 and mAb2C2 also detected a band of 93 kDa, which was detected with mAb1D9. Consistent with this result, IPs from the control serum were not recognized by mAb2C2 or mAb1D9.

The 93 kDa bands were subjected to mass spectrometric analysis followed by a database search using the *T. cruzi* CL Brener (hybrid clone) genome database (**Supplementary Table S1**). The mAb2C2 immunoprecipitates from the CL strain showed high identity with sequences from two CL Brener haplotypes, namely, TcCLB.507089.170 (Esmeraldo-like haplotype) and TcCLB.506725.20 (non-Esmeraldo-like haplotype), with 16% coverage. Both sequences code for the same 67 kDa hypothetical protein. Interestingly, mass spectrometric analysis of the 93 kDa band from the immunoprecipitation of the G strain with mAb2C2 and mAb1D9 also identified the same protein (TcCLB.506725.20) with 19.6 and 17.2% coverage, respectively (**Supplementary Table S1**). Proteins identified by mass spectrometry in the IP of the G strain are listed in **Supplementary Table S2**; TcCLB.506725.20 had the highest score among the proteins.

*In silico* analysis predicted an N-terminal signal peptide with high probability, indicating that this protein is secreted (**Figure 3B** and **Supplementary Figure S1**). An enzymatic domain with alpha/beta hydrolase activity, hydrophobic domains in the N- and C-terminal regions, six potential *N*-glycosylation sites, and eight potential *O*-glycosylation sites were also predicted by bioinformatics. Although this protein does not have a classic glycosylphosphatidylinositol (GPI)-anchor domain, a highly hydrophobic C-terminal domain of approximately 20 amino acids is predicted, and without this terminal domain, Ssp-4 has 100% probability of being anchored to GPI.

The immunogenic peptides of the predicted protein were assessed by prediction of the tertiary structure of the protein using homology modeling and threading by two different algorithms (I-Tasser and Yasara) (**Figure 3C**). In these models, the peptides with higher immunogenic potential from the protein are displayed in different colors (blue, green, red, and yellow). From the structural analysis, peptides LREFVRSTERNR (named MAP2, shown in yellow) and EARDQQALTQLR (shown in green) predicted to be more exposed on the protein surface compared to peptides LNWGADAKEMYTEYR (shown in blue) and VGENFDDSWASDLRR (shown in red) (**Supplementary Table S3**). Only MAP2 was selected for polyclonal antibody production in mice. The polyclonal antibody against the MAP2 peptide reacted with a band of 93 kDa from EA lysates immunoprecipitated with mAb2C2 (**Figure 3E**), confirming that the MAP2 peptide is part of the protein core of Ssp-4 (**Figure 3D**).

**FIGURE 4:**
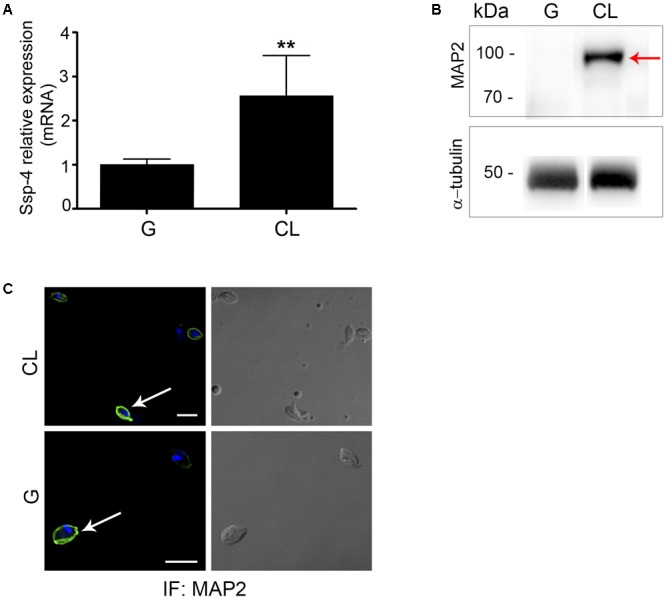
Ssp-4 protein expression is increased in the less infective strain. **(A)** Relative mRNA expression of Ssp-4 from extracellular amastigotes (EAs) of the CL strain compared to EAs from the G strain in three independent experiments performed in triplicate. ^∗∗^*p* < 0.01. **(B)** Whole cell lysates (WC) of EAs of the G and CL strains were incubated with MAP2 in Western blotting; anti-α-tubulin antibody was used for normalization. Red arrows indicate Ssp-4. **(C)** Immunofluorescence (IF) of EAs of the G and CL strains incubated with MAP2 (green); nuclei and kinetoplasts were labeled with DAPI (blue). Scale bar: 5 μm.

These data confirm that the hypothetical protein identified by mass spectrometry is Ssp-4, as previously identified by mAb2C2 (Andrews et al., 1988). We sequenced nucleotides from the *T. cruzi* CL Brener, G and CL strains, and the protein alignment revealed that the Ssp-4 sequence is well conserved across all strains and among CL Brener haplotypes, clone Dm28c, and the Sylvio strain from the genome database (**Supplementary Figure S2A**). Protein identity between all strains was analyzed and was found to be at least 95.7% (**Supplementary Figure S2B**). The identified Ssp-4 sequence allowed us to compare the mRNA expression of Ssp-4 in infectious and less-infectious EA strains; we observed that Ssp-4 mRNA expression, measured by qPCR, is at least twofold higher in less-infectious strains compared to infectious EAs (**Figure 4A**). This difference was confirmed by Western blotting and immunofluorescence with a MAP2 antibody (**Figures 4B,C**). Therefore, the invasiveness of infectious strains is not associated with Ssp-4 mRNA or protein expression. These data suggest that Ssp-4 glycosylation and carbohydrate epitopes recognized by mAb1D9 could be related to EAs infectivity.

### EAs Secrete Glycosylated Ssp-4 That May or May Not Be Associated With Vesicles

Because *in silico* analysis identified a signal peptide on the Ssp-4 protein core (**Figure 3B** and **Supplementary Figures S1, S2**), we assessed the secretion of the glycosylated form of Ssp-4 (detected by mAb1D9) using confocal and electron microscopy and SDS–PAGE/silver staining on EA supernatant fractions.

**FIGURE 5:**
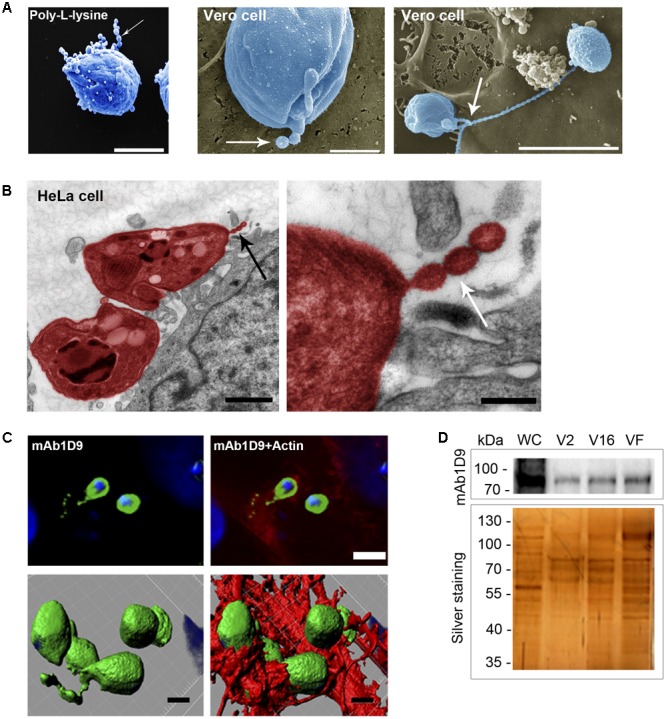
Extracellular amastigotes release Ssp-4 associated with vesicles interacting with host cell. **(A)** Scanning electron microscopy of extracellular amastigotes (EAs) adhered to coverslips coated with poly-L-lysine or Vero cells. Scale bars: 2 and 5 μm. **(B)** Transmission electron microscopy of HeLa cells incubated for 30 min with EAs from the G strain (colored in red). Scale bar: 1 μm. Black arrows indicate EA secreted vesicles. **(C)** Immunofluorescence of HeLa cells incubated for 30 min with EAs from G (MOI 10:1) and mAb1D9 (green), DAPI (blue), and phalloidin-TRITC (red). Upper panels: images of a focal plane showing vesicles secreted by EAs of the G strain (green) associated with actin (red); bottom panels: three-dimensional reconstruction from a *Z*-series acquired by a confocal microscope. Scale bar: 2 μm. Arrows indicate vesicular structures secreted by EAs. **(D)** Extracellular amastigotes (EAs) from the G and Y strains were incubated for 6 h in RPMI medium without fetal bovine serum. Supernatant was collected and fractionated in three different populations: V2 (pelleted after 2 h of ultracentrifugation), V16 (pelleted after 16 h ultracentrifugation), and VF (supernatant from pellet V16). Western blotting with mAb1D9 and silver-stained SDS–PAGE of the different fractions. Whole cell (WC) lysate of EAs of the G strain was used as control.

First, morphological analysis of the microscopy images revealed that infectious EAs release vesicles to the extracellular medium, and these vesicles are either attached to poly-L-lysine-coated substrates or to host cell surfaces (**Figures 5A,B**). Vesicles of regular shape (100–200 nm in diameter) are secreted in trails (**Figures 5A,B**, arrows). These vesicle trails display the carbohydrate epitope recognized by mAb1D9 and are shed locally at sites of close contact between EAs and host cells, characterized by phagocytic cups formed by host cell actin (**Figure 5C** and **Supplementary Figure S3**).

**FIGURE 6:**
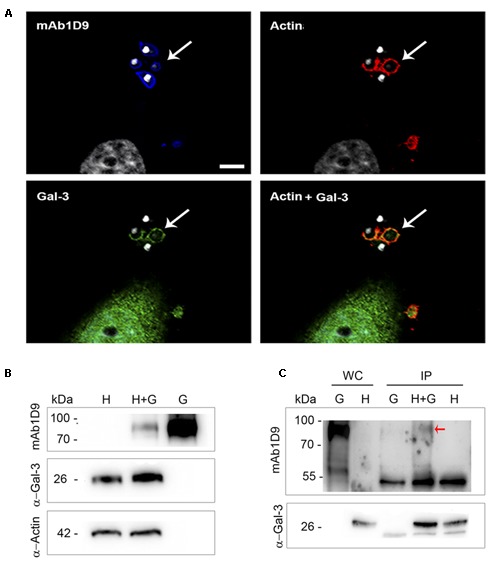
Galectin-3 is recruited to extracellular amastigotes from the G strain at sites of invasion and interacts with the Ssp-4 carbohydrate epitope recognized by mAb1D9. **(A)** Immunofluorescence with mAb1D9 (blue) and anti-Gal-3 (green). HeLa cells were incubated with extracellular amastigotes (EAs) from the G strain (MOI 10:1) for 30 min. The actin cytoskeleton was labeled with 647 phalloidin (red), and DAPI (white) was used to label nuclei and kinetoplasts. White arrows indicate EAs surrounded by actin and Gal-3. Scale bar: 5 μm. **(B)** Western blotting of EAs interacting with HeLa cells performed with anti-Gal-3 and mAb1D9. Samples were normalized with anti-α-actin. HeLa cells were incubated with EAs of the G strain (MOI 40:1) for 40 min. Whole cell (WC) lysate from infected cells (H+G), and controls only from HeLa (H) and EAs of the G strain (G) were obtained. **(C)** Immunoprecipitation (IP) was performed for H+G and controls of H and G lysates with anti-Gal-3 coupled to protein G-Sepharose. Red arrow indicates the band that reacted with mAb1D9 on the IP H+G.

These phagocytic cups associated with the interactions of infectious EAs are also rich in galectin-3 (Gal-3); **Figure 6A** shows that the actin of the phagocytic cup and Gal-3 colocalize in regions of interaction between host cells and infectious EAs tagged with mAb1D9. The host cell origin of Gal-3 was confirmed by Western blotting on extracts of host cells or EAs alone and of host cells interacting with EAs (**Figure 6B**). Additionally, host cells infected by EAs present 20% more Gal-3 than non-infected host cells (**Figure 6B**). The direct interaction between the Ssp-4 carbohydrate epitope recognized by mAb1D9 and host cell Gal-3 was assessed by immunoprecipitation using anti-Gal-3; **Figure 6C** shows that only the lysates of infected host cells presented reactivity with mAb1D9 (red arrow), indicating that the carbohydrate epitope recognized by mAb1D9 and displayed on Ssp-4 is a β-galactoside, with which Gal-3 is known to specifically interact (Hughes, 1999).

After demonstrating that the carbohydrate moiety of Ssp-4 is a β-galactoside that can be recognized by host cell surface components and is secreted in vesicles during interactions with host cells at sites of EA invasion, we evaluated whether glycosylated Ssp-4 could also be found in vesicle-free fractions of infectious EA supernatants (**Figure 5D**). Fractions were obtained by ultracentrifugation of the EA supernatant after 2 or 16 h, resulting in precipitates containing ectosomes (V2 fraction) and exosomes (V16 fraction), respectively. The remaining supernatant is considered free of vesicles (VF fraction). **Figure 5D** shows that although SDS–PAGE/silver staining demonstrated different protein profiles between supernatant populations, glycosylated Ssp-4 was detected by mAb1D9 in all three fractions, demonstrating that infectious EAs release either free glycosylated Ssp-4 or glycosylated Ssp-4 in association with different secreted vesicles. No differences in electrophoretic mobility were observed between vesicle-free and vesicle-associated Ssp-4 protein. This finding is consistent with previous observations about the *T. cruzi* secretome from metacyclic typomastigotes, where the stage-specific GPI-anchored protein GP82 detected in vesicle-free fractions had a mobility profile that matched that of the protein present in vesicle-enriched fractions (Bayer-Santos et al., 2013).

## Discussion

Mortara et al. (1999) first established correlation between Ssp-4 recognition by mAb1D9 and EA infectivity by demonstrating that EAs of more infective strains present higher reactivity with mAb1D9. However, da Silva et al. (2006) did not observe the same correlation between mAb1D9 reactivity and EA infectivity in isolates from chagasic patients. Although correlation was not demonstrated, the authors verified that blockage of the carbohydrate epitope with mAb1D9 could inhibit EA invasion, suggesting a role for carbohydrate moieties of Ssp-4 in host cell invasion. We confirmed the correlation between the Ssp-4 carbohydrate epitope recognized by mAb1D9 and EA infectivity, adding these observations to those from the sequencing of the Ssp-4 protein core, the measurement of the expression of this protein in infectious versus less-infectious EAs and the analysis of the nature and function of the carbohydrate moieties in parasite infectivity.

The EAs from the most infective strains, G and clone Tc1552, which belong to the same phylogenetic group (Tc I), exhibited high reactivity with mAb1D9 and low reactivity with mAb2C2 (**Figures 1, 2**). Conversely, less-infectious EAs, such as those of the CL strain (Tc VI) and the Tc863 isolate (Tc III), showed high reactivity with mAb2C2 and low reactivity with mAb1D9. These results suggest that the post-translational modifications of Ssp-4 differ among *T. cruzi* strains, and this phenotype directly reflects parasite infectivity.

The first description of Ssp-4 revealed that mAb2C2 recognizes a surface GPI-anchored glycoprotein, which is cleaved and released into extracellular milieu in the presence of PI-PLC (Andrews et al., 1988). Subsequently, Olivas-Rubio et al. (2009) identified a partial sequence from the DGF-1 multigenic family, but identification of potential domains on the putative protein was challenging. These challenges led us to identify Ssp-4 using approaches other than immunostaining, such as mass spectrometry (MS) of mAb2C2- and mAb1D9-immunoprecipitated proteins, protein sequencing with bioinformatic analysis and quantitative PCR.

Based on the CL Brener hybrid clone genomic database, haplotype sequences encode a hypothetical protein that is highly expressed (mRNA and protein) in amastigote forms (Atwood et al., 2005; Minning et al., 2009). Additionally, Ssp-4 sequences exhibit homology with a hypothetical protein from *Trypanosoma rangeli* and *Leishmania* ssp., but not *Trypanosoma brucei*, which indicates that these proteins could be associated with the presence of an intracellular cycle defined by the amastigote form. *In silico* analysis of the amino acid sequences identified by MS led to the identification of Ssp-4 domains previously described by Andrews et al. (1988) as potential sites to be targeted to the plasma membrane and secreted, i.e., the presence of *N*- and *O*-glycosylation sites and a possible GPI-anchor. Potential glycosylation sites observed explain the fact that hypothetical protein encountered by MS present 67 kDa, and by Western blotting molecular weight detect a band between 93 kDa. Finally, an α/β-hydrolase motif was identified. Previous study found that the trypomastigote surface prolyl oligopeptidase (POP) enzyme (Tc80), which contains an α/β-hydrolase domain, is capable of hydrolyzing substrates such as fibronectin and collagen and was shown to be involved in non-phagocytic cell invasion (Bastos et al., 2005). However, the enzymatic functions of this feature of Ssp-4 were not investigated, and the contribution of this enzyme to host cell invasion remains unclear.

To confirm our MS data, a polyclonal antibody (MAP2) was produced based on the tertiary structure of Ssp-4 (**Figure 3C**). In this set of results, it was observed that MAP2 reacts with mAb2C2 immunoprecipitates (mAb2C2-IPs) from less infectious EAs (**Figure 3D**). These data strongly suggest that the protein detected by mass spectrometry is the Ssp-4 that was previously described by Andrews et al. (1988). Immunostaining using the MAP2 antibody detected higher expression of Ssp-4 on non-infectious EAs, suggesting again that recognition of the carbohydrate epitope on Ssp-4 by mAb1D9 can hinder the interactions of the protein core epitopes (**Figure 4B**). The production of the MAP2 antibody, obtained after chemical synthesis of peptides generated by bioinformatics and protein modeling, confirms the antigenic potential of Ssp-4 and renders feasible the production of an efficient and safe vaccine against Chagas’ disease using a combination of the Ssp-4 protein core and carbohydrate moieties (Quijano-Hernandez and Dumonteil, 2011; Ma et al., 2015).

The hypothetical protein (TcCLB.506725.20) identified by MS and considered here to be Ssp-4 was previously detected by our group in microarray assays. In the previous experiments, a 21-kDa protein (P21) and a mevalonate kinase (MVK) were also detected, being more highly expressed in the infectious EAs of the G strain than in the less-infectious CL strain; these two factors were shown to contribute to host cell invasion (da Silva et al., 2009; Ferreira et al., 2016). Here, we confirmed by quantitative PCR that Ssp-4 is more highly expressed in less-infectious strains (**Figure 4A**), although infectious EAs exhibit a highly glycosylated Ssp-4 (as revealed by mAb1D9). Thus, we concluded that even though Ssp-4 provides the substrate for other posttranslational modifications, host cell invasion is not determined by Ssp-4 protein expression alone but by glycosylation of Ssp-4 (**Figure 4**).

Considering that glycosylated Ssp-4 is secreted and that infectious EAs secrete large amounts of vesicle trails when adhered to poly-L-lysine surfaces covered by glycosylated Ssp-4 or mammalian host cell surfaces (**Figures 5A,B**) (Barros et al., 1996), we investigated whether or not Ssp-4 interacted with these vesicles to modulate EA infection by either inhibiting or promoting host cell invasion. A recent study observed a similar pattern of secreted vesicles from flagellar membranes of *Trypanosoma brucei* that act as carriers of virulence factors, contributing to host immune evasion and erythrocyte remodeling (Szempruch et al., 2016). First, vesicles displaying glycosylated Ssp-4 are secreted locally at sites of EA-host-cell interaction, namely, the phagocytic cup formed during the process of EA internalization, suggesting that these vesicles could be recognized by host cell receptors and could ultimately promote host cell invasion. Therefore, we sought to identify a possible receptor that could bind to carbohydrates displayed by Ssp-4. Several groups have demonstrated that Gal-3 participates in host cell infection by *T. cruzi*; Machado et al. (2014) observed the recruitment of Gal-3 during invasion and after parasitic escape from vacuoles in peritoneal macrophages infected with EAs of the G strain. Considering that EAs are internalized by non-professional phagocytes via an actin-dependent process similar to phagocytosis (Mortara, 1991; Procópio et al., 1998; Fernandes et al., 2013) and that Gal-3 has been shown to play a crucial role during phagocytosis in macrophages (Sano et al., 2003), we investigated the association of Gal-3 with the Ssp-4 carbohydrate epitope recognized by mAb1D9 during host cell invasion by EAs. We have demonstrated that Gal-3 is recruited to the EA entry site (**Figure 6A**), is more abundant in infected cells than in uninfected cells (**Figure 6B**) and co-immunoprecipitates with Ssp-4 recognized by mAb1D9 (**Figure 6C**). As Gal-3 specifically recognizes β-galactosides, we concluded that a β-galactose antigen displayed by Ssp-4 is the carbohydrate moiety recognized by host cells. Exogenous Gal-3 is able to activate Rac-1 in corneal epithelial cells (Saravanan et al., 2009). Interestingly, our group has previously revealed the participation of proteins associated with the Rho-GTPase family, such as Rac-1, in EA internalization (Fernandes and Mortara, 2004; Bonfim-Melo et al., 2018). Members of these families are responsible for mediating the recruitment of the actin cytoskeleton with cell surface receptors. Thus, increased Gal-3 availability could mediate Rac-1 activation and formation of phagocytic cups involved in host cell invasion of EAs.

We have shown that post-translational modifications of surface proteins of *T. cruzi* EAs are more closely associated with parasite infectivity than the expression of the protein core. Additionally, glycosylated Ssp-4 can form a bridge between the parasite and host cell surface factors such as Gal-3, triggering EA internalization. In our model, we have shown that protein glycosylation plays a central role in the control of EA host cell invasion, highlighting the importance of post-translational modifications. The results of this study may contribute to the development of therapeutic agents and vaccines for the treatment of Chagas’ disease.

## Materials and Methods

### Host Cells

HeLa cells and Vero cells (Instituto Adolfo Lutz, São Paulo, Brazil) were cultivated by successive passaging in RPMI 1640 medium (Atena Biotecnologia) supplemented with 10% heat-inactivated fetal bovine serum (FBS; Vitrocell), 100 U/mL penicillin and 100 μg/mL streptomycin (Sigma-Aldrich).

### Parasites

In this study, parasites from the G (Tc I) (Yoshida, 1983) and CL (Tc VI) strains (Brener and Chiari, 1963) were used. New isolates of *T. cruzi* were also used: the Tc863 (Tc III) isolate and clones Tc1522 (José strain; Tc I) and Tc1994 (Tcbat). These previously classified/typed isolates were a gift from Dr. Marta M. Geraldes Teixeira (ICB-USP, Trypanosomatid Bank). *T. cruzi* trypomastigote forms were collected from the supernatants of Vero cells cultivated with RPMI medium supplemented with 2.5% heat-inactivated FBS. The trypomastigotes were then incubated with liver infusion tryptose (Camargo, 1964) medium (pH 5.8) supplemented with 10% FBS for 16 h to obtain EAs (Tomlinson et al., 1995).

### Ssp-4 Antibodies

Monoclonal antibody (mAb) 2C2, which recognizes an epitope of Ssp-4, was a gift from Norma Andrews (University of Maryland) (Andrews et al., 1987). The antibody mAb1D9 was obtained by immunization of BALB/c mice with amastigotes from clone D11 (derived from G strain).

Peptides (Bio-Synthesis Inc.) were synthesized based on the Ssp-4 tertiary structure modeled by homology modeling and threading (described in section “Molecular Modeling and Structural Analyses”). We selected non-conjugated multiple antigenic peptide sequence (LREFVRSTERNR) for animal immunization. Briefly, solubilized peptides (1 mg/mL) in phosphate-buffered saline (PBS; 137 mM NaCl, 10 mM phosphate, 2.7 mM KCl, pH 7.4) were inoculated into BALB/c mice in the presence of Freund’s complete or incomplete adjuvant (Sigma-Aldrich). Four immunizations (100 μL peptide solution/mouse) were performed intraperitoneally with 7-day interval. These mice were euthanized with lethal injections of ketamine/xylazine (250/50 mg/kg per mouse) 1 week after the last immunization. Then, cardiac puncture was performed on these mice, and the collected blood was centrifuged at 1,200 × *g* for 5 min to obtain serum containing polyclonal antibodies (MAP2). Control serum was obtained as a negative control by caudal vein puncture before the first immunization. All animal procedures were approved by the local ethics committee (No. 1465020616).

### Immunoprecipitation

Ssp-4 was immunoprecipitated (IP) using protein A-Sepharose (Sigma-Aldrich) with mAb2C2 (IgG2a). Lysate was prepared from 10^9^ EAs of the CL or G strains with lysis buffer (50 mM Tris–HCl, 150 mM NaCl, 1% Triton X-100, pH 7.4). The lysate was then incubated with protein A-Sepharose for 1 h at 4°C for pre-clearing. Next, the supernatant from the pre-clearing was incubated with protein A-Sepharose and mAb2C2 (1:5, ascitic fluid) overnight at 4°C. After incubation, the pellet containing protein A-Sepharose attached to mAb2C2 and Ssp-4 was washed with washing buffer (20 mM Tris–HCl, pH 7.5) with different NaCl concentrations (0.5, 0.3, 0.1 M). The pellet was then resuspended with 4 × SDS–PAGE sample buffer (0.1 M Tris–HCl, 10% SDS, 0.5 mM bromophenol blue, 10% glycerol, 12% β-mercaptoethanol, pH 6.8). As a control, we used a non-immune mouse serum for immunoprecipitation instead of mAb2C2. Alternatively, immunoprecipitation was performed using EAs from the G strain lysate with mAb1D9 (IgG3) and protein G-Sepharose (Sigma-Aldrich).

### Mass Spectrometric Analysis

The IPs from the EAs of the CL strain with mAb2C2 and the control serum (CS) and IPs from the EAs of the G strain with mAb2C2, mAb1D9, and CS were subjected to SDS–PAGE and stained with colloidal Coomassie. The band of 93 kDa from all mAb2C2 and CS IPs were excised from the gel and destained with 50 mM ammonium bicarbonate in 50% acetonitrile (ACN) solution. Then, the bands were dehydrated with 100% ACN. Proteins were reduced with 10 mM dithiothreitol and alkylated with 50 mM iodoacetamide. Gel bands were washed with 50 mM NH_4_HCO_3_/50% ACN and then with 100% ACN. The bands were then incubated with porcine trypsin (Sigma-Aldrich) solution (400 ng) for 16 h at 37°C for protein digestion. The resulting peptides were resuspended in 0.1% formic acid, and an aliquot (4.5 μL) was injected into a Q-TOF Ultima mass spectrometer (Waters) coupled to a nano-chromatography system (nanoACQUITY, Waters). Samples were desalted and concentrated using a pre-column (Symmetry C18, 180 μm × 20 mm, Waters) and eluted onto a capillary reversed-phase C18 column (10 cm × 75 μm, Waters) at a flow rate of 600 nL/min. Peptides were fractionated with a linear gradient from 5 to 40% ACN in 0.1% formic acid for 15 min. The eluted peptides were directly injected into the mass spectrometer (MS) via an electrospray. The MS spectrum of phosphoric acid was acquired simultaneously to acquisition of the sample spectra. The MS/MS spectra of the three most intense peaks with two to four charges in the MS function were automatically acquired in the data-dependent acquisition (DDA) mode. The capillary voltage and reference cone were set at 3.2 kV and 100 V, respectively. All analyses were performed in positive ion mode. The spectra were acquired from 50 to 2000 m/z.

Peptides from the IPs of the G strain were injected onto an LTQ-Orbitrap Velos (Thermo). Masses from the raw Q-TOF files were corrected using a lock mass of m/z 784.823, and the raw data were converted to the .pkl format using the ProteinLynx Global Server (Waters). Orbitrap raw files were converted into .mgf files using MSConverter. Searches were performed in MASCOT (2.4.1 version) against a *Trypanosoma cruzi* CL Brener database (downloaded in 2017/05, 21,169 sequences) by considering a peptide and fragment mass tolerance of 0.5 Da (for Q-TOF files) and peptide and fragment mass tolerance of 10 ppm and 0.6 Da, respectively. Carbamidomethylation of cysteine and oxidation of methionine residues were specified as fixed and variable modifications, respectively. To obtain a final list of identified proteins, masses smaller than 60 kDa and higher than 110 kDa were excluded as were proteins identified as control samples. Finally, proteins identified with scores lower than 80 were also excluded.

### *In Silico* Analysis

Bioinformatics was performed using the ExPASy^1^ platform. The signal peptide was predicted by SignalP v. 4.1. The TargetP v. 1.1 platform was used to predict subcellular location. Hydrophobic domains were predicted by the TMHMM 2.0 server. Potential sites of *N*- and *O*-glycosylation were identified by NetNGlyc 1.0 and NetOGlyc 4.0, respectively. Interproscan v. 4.8 software was used to identify different protein domains. Prediction of GPI-anchoring sites was performed with the PredGPI^2^ program.

### Cloning of the Ssp-4 Coding Sequence

The open reading frame encoding the putative Ssp-4 protein identified by mass spectrometry was amplified by PCR using *T. cruzi* genomic DNA as a template. The amplification was performed using the Ssp4F (5′-atggctctaaagagtatgcggaaa-3′) and Ssp4R (5′-ttaatggttcaaattgctgtaaaa-3′) oligonucleotides and *Pfu* DNA polymerase (Fermentas).

Amplicons from the G and CL strains and the CL Brener clone were resolved on agarose gels, and the bands at the expected size were excised and purified. Purified amplicons were A-tailed prior to ligation into the pGEM^®^-T Easy vector (Promega). Plasmid DNA isolated from positive clones was sequenced on an ABI3500 genetic analyzer (Applied Biosystems) using the BigDye^®^ Terminator v3.1 Cycle Sequencing Kit (Life Technologies). Sequences were aligned by BioEdit v. 7.2.5 using the ClustalW algorithm. The assembled nucleotide sequences encoding the putative Ssp-4 protein from the G and CL strains were deposited in GenBank under accession numbers KX581696 and KX581697, respectively.

### Relative Quantification of mRNA by Real-Time PCR (qPCR)

For real-time quantitative PCR (qPCR), we synthesized the following primers: GAPDH Forward (5′-agcgcgcgtctaagacttaca-3′) and Reverse (5′-tggagctgcggttgtcatt-3) (Cordero et al., 2008) and Ssp-4 Forward (5′-cctctgacattgacccgttatt-3′) and Reverse (5′-gtaagttggattggtgtggta-3′). RNA extraction from the EAs of the G and CL strains was performed with the RNeasy Protect Mini Kit (Qiagen). Next, cDNA was generated using the High-Capacity cDNA Reverse Transcription Kit (Applied Biosystems). Relative quantification was monitored by intercalation of SYBR^TM^ Green Master Mix (Life Technologies) in the double-stranded DNA in qPCR reactions; normalized values calculated with a ΔΔcycle threshold (Ct) and relative expression (2^-ΔΔC_t_^) with the G strain as a reference are shown. GAPDH is the housekeeping gene; this gene is constitutively expressed in *T. cruzi*.

### Molecular Modeling and Structural Analyses

The three-dimensional structure of the putative Ssp-4 sequenced by mass spectrometry was modeled using I-Tasser (Yang et al., 2015) and Yasara^3^. The model with the highest *C*-score from I-Tasser and the hybrid model from Yasara were selected for structural analysis. The solvent-accessible surface area (SASA) of each amino acid was calculated using DSSP 2.2.1 (Touw et al., 2015). The most exposed peptides in both models were selected as the most immunogenic peptides to be synthesized (Bio-Synthesis Inc.) for the production of the antibody against the Ssp-4 protein region (section “Ssp-4 Antibodies”).

### Western Blotting

The EA lysates (20 μg) were incubated with 4 × sample buffer and subjected to SDS–PAGE (Laemmli, 1970). Proteins from the gel were transferred to nitrocellulose membranes to perform the immunoblotting (Towbin et al., 1979). Membranes were blocked for 1 h with ECL blocking agent (GE Healthcare), TBS (50 mM Tris–HCl, 150 mM NaCl, pH 7.4), and TBS-T (TBS with 0.1% Tween 20). Then, nitrocellulose membranes were incubated with mAb1D9 (1:500, ascites), mAb2C2 (1:500 ascites), MAP2 (1:500, mouse antiserum), or anti-α-tubulin (1:1000, Sigma-Aldrich) overnight with 5% bovine serum albumin (Sigma-Aldrich) in TBS-T. Next, the membranes were incubated for 1 h with a secondary antibody, anti-mouse IgG-peroxidase (Sigma-Aldrich), and visualized by a chemiluminescence detector (UVITEC) in the presence of luminol with the ECL Prime Western blotting detection reagent (GE Healthcare).

### Transmission and Scanning Electron Microscopy

HeLa cells incubated with EAs for 30 min were fixed with 2.5% glutaraldehyde and 2% formaldehyde in 0.1 M sodium cacodylate buffer at room temperature for 1 h and then processed for transmission electron microscopy (TEM) according to procedures described elsewhere (Arruda et al., 2012). Cells were observed in a JEOL 1200 EXII electron microscope (JEOL Ltd., Peabody, MA, United States). For scanning electron microscopy (SEM), EAs adhered to Vero cells or to coverslips covered with 0.1% poly-L-lysine (Sigma-Aldrich) were fixed with 4% paraformaldehyde, washed with 0.1 M cacodylate solution, post-fixed with osmium tetroxide, treated with tannic acid, dehydrated with ethanol, dried in a critical point dryer (Bal-Tec AG, Balzers, Liechtenstein), and sputtered with gold. Samples were observed in an FEI Quanta FEG 250 SEM (Hillsboro, OR, United States).

### Parasites Immunofluorescence and Confocal Imaging

The EAs were attached to coverslips covered with 0.1% poly-L-lysine (Sigma-Aldrich) for 50 min or HeLa cells for 30 min at 37°C and fixed with 4% paraformaldehyde. After fixation, coverslips were incubated with blocking solution, PGN (0.2% gelatin, 0.1% NaN_3_, diluted in PBS), for 1 h. Next, samples were incubated with mAb1D9, mAb2C2 (1:100), or MAP2 (1:100) diluted in PGN supplemented with 0.25% saponin (PGN-S; Sigma-Aldrich) for 1 h at room temperature. Next, coverslips were incubated with a secondary antibody, Alexa Fluor 488 IgG-mouse (Invitrogen), diluted in PGN-S (1:100) for 1 h at room temperature. Coverslips were then incubated with 1 μM 4′,6-diamidino-2-phenylindole (DAPI) for 15 min. After three washes with PBS, coverslips were mounted in glycerol buffered with 0.1 M Tris (pH 8.6) and 0.1% *p*-phenylenediamine. Samples were examined by a Leica TCS SP5 confocal system (Leica Microsystems, Wetzlar, Germany) using a 100 × NA 1:44 oil immersion objective. Images were processed by Imaris software (Bitplane AG, Andor Technology, Belfast, United Kingdom).

### EA Supernatant Fractioning

The EAs supernatant fractionation was adapted from Bayer-Santos et al. (2013). Briefly, parasites from the G strain were incubated in RPMI medium without FBS for 6 h at 37°C (10^8^ EAs/mL). Then, the parasites were removed by centrifugation at 3,000 × *g* for 10 min, and supernatant was collected. The supernatant was filtered by using a 0.45-μm-pore membrane and subjected to ultracentrifugation at 100,000 × *g* for 2 h to obtain the first pellet, which was enriched with larger vesicles (V2). The supernatant resulting from this ultracentrifugation was again subjected to ultracentrifugation at 100,000 × *g* for 16 h to generate the second pellet, which was enriched with smaller vesicles (V16), and the supernatant contained vesicle-free soluble (VF) proteins.

### Infection of Host Cells

Suspension containing 10^5^ HeLa cells in RPMI supplemented with 10% FBS per well (24-well plate) was plated on coverslips and incubated overnight at 37°C in CO_2_ (5%). Next, EAs (Multiplicity of Infection; MOI: 10:1) were added for 2 h at 37°C. After incubation, the cells were washed 5–7 times with PBS. Then, the cells were fixed with Bouin’s solution (Sigma-Aldrich) for 5 min and stained with Giemsa for 1 h (da Silva et al., 2006). Internalized parasites were quantified by light microscopy (Bonfim-Melo et al., 2015).

Alternatively, EAs were added to HeLa cells adhered to 6-well plates and incubated for 40 min at 37°C in CO_2_ (5%). An immunofluorescence assay was performed on the coverslips; after EA (MOI 10:1) incubation, the cells were washed with PBS and fixed with 4% paraformaldehyde (PFA) for 15 min. Then, the coverslips were incubated with the primary antibodies mAb1D9 (mouse IgG) and anti-Galectin-3 (rat IgG; a gift from Dr. Roger Chammas, Universidade de São Paulo) for 1 h at room temperature. Next, the samples were incubated with the secondary antibodies Alexa Fluor IgG mouse 488 and Alexa Fluor IgG rat 568 in the presence of 1 μm DAPI (Invitrogen) and 200 units/mL phalloidin 647 (Invitrogen) for 1 h at room temperature.

For Western blotting, EAs (MOI 40:1) were incubated with HeLa cells for 40 min at 37°C in CO_2_ (5%). Infected HeLa cells, control non-infected HeLa cells, and EA suspensions were lysed with lysis buffer (50 mM Tris–HCl, 150 mM NaCl, 1% Triton X-100, pH 7.4) for 30 min at 4°C. Samples were subjected to Western blotting with primary antibodies anti-Gal-3 (1:250), mAb1D9 (1:500), and anti-α-actin (1:1000; Cell Signaling), and then, immunoprecipitation with anti-Gal-3 and protein G-Sepharose (Sigma-Aldrich) was performed as described above. Band densitometry was analyzed by UVIBAND image quantification software.

### Statistics

All results are representative of three independent experiments with at least two biological replicates. Excel and GraphPad Prism 4.0 were employed for data plotting and statistical analysis. Statistical tests included Student’s *t*-test and ANOVA, and a statistical threshold of *p* < 0.05 was used.

## Author Contributions

PF, FR, CO, and RM designed and coordinated the study and wrote the paper. JdC, PF, and FV performed the proteomic experiments and analyses. EC, PF, and CO designed and performed DNA sequencing experiments. TS designed the three-dimensional structure analysis and peptide selection for antibody production. All authors analyzed the results and approved the final version of the manuscript.

## Conflict of Interest Statement

The authors declare that the research was conducted in the absence of any commercial or financial relationships that could be construed as a potential conflict of interest.

## Footnotes

1 www.expasy.org

2 http://gpcr.biocomp.unibo.it/predgpi/pred.htm

3 www.yasara.org

## Supplementary Material

FIGURE S1 *In silico* analysis of the Ssp-4 sequence. Analysis of the Ssp-4 protein sequence identified by mass spectrometry using the ExPASy platform. The results obtained from the server are as follows: **(A)** prediction of signal peptide (SignalP v. 4.1); **(B)** prediction of transmembrane helices (TMHMM v. 2.0); **(C)** prediction of GPI-anchor (PredGPI); **(D)** prediction of subcellular localization (TargetP v. 1.1); **(E)** Interproscan v. 4.8; **(F)** prediction of *N*-glycosylation (NetNGlyc v. 1.0); and **(G)** prediction of *O*-glycosylation (NetOGlyc v. 4.0).

FIGURE S2 Ssp-4 protein alignment revealed a conserved backbone between different *T. cruzi* strains. **(A)** Sequences were aligned in the BioEdit program using the ClustalW algorithm. Regions with 100% identity between the sequences are shown in black. White and gray regions represent amino acid divergence in at least one of the aligned sequences. In the legend, 1 and 2 represent the Esmeraldo-like haplotype and non-Esmeraldo-like haplotypes from the CL Brener database, respectively; 3 and 4 represent Dm28c and Sylvio database sequences, respectively; 5, 6 and 7 represent Ssp-4 coding sequences from the CL Brener clone and the G and CL strains isolated in this study, respectively. **(B)** Protein identity and divergence scores between all strains aligned.

FIGURE S3 Vesicle trails covered with Ssp-4 carbohydrate epitopes on EAs of the G strain adhered to poly-L-lysine. Extracellular amastigotes (EAs) were attached onto coverslips coated with poly-L-lysine for 50 min at 37°C. Then, the parasites were fixed with 4% paraformaldehyde and incubated with blocking solution for 1 h. Samples were incubated with mAb1D9 (green) and DAPI (blue). Left panels: immunofluorescence images obtained from one plane. Arrows indicate released vesicle trails from parasites. Right panels: Differential interference contrast (DIC). Scale bar: 2 μm.

TABLE S1 Proteins and peptides identified by mass spectrometry.

TABLE S2 List of identified proteins from EAs of the G strain immunoprecipitated with mAb2C2 and mAb1D9.

TABLE S3 Solvent-accessible surface area (SASA). The solvent-accessible surface area (SASA) for each amino acid predicted by DSSP 2.2.1.
