# Supplementary figures and images for: A Carbohydrate Moiety of Secreted Stage-Specific Glycoprotein 4 Participates in Host Cell Invasion by Trypanosoma cruzi Extracellular Amastigotes
Source: Front Microbiol. 2018 Apr 10;9:693. doi: 10.3389/fmicb.2018.00693 (PMC5903068; doi:10.3389/fmicb.2018.00693)

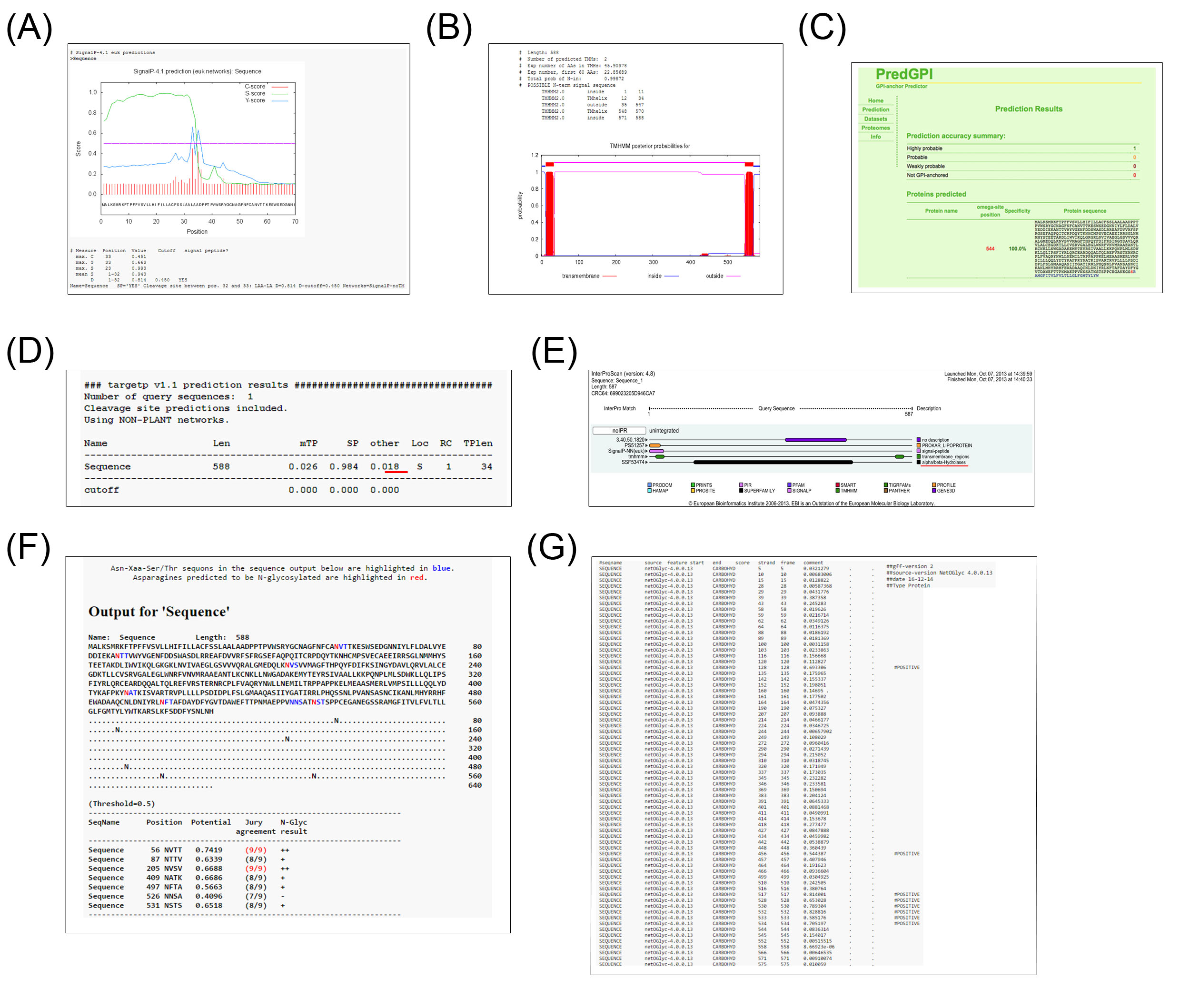

Supplement: FIGURE S1 — In silico analysis of the Ssp-4 sequence. Analysis of the Ssp-4 protein sequence identified by mass spectrometry using the ExPASy platform. The results obtained from the server are as follows: (A) prediction of signal peptide (SignalP v. 4.1); (B) prediction of transmembrane helices (TMHMM v. 2.0); (C) prediction of GPI-anchor (PredGPI); (D) prediction of subcellular localization (TargetP v. 1.1); (E) Interproscan v. 4.8; (F) prediction of N-glycosylation (NetNGlyc v. 1.0); and (G) prediction of O-glycosylation (NetOGlyc v. 4.0). [file Image_1.JPEG]

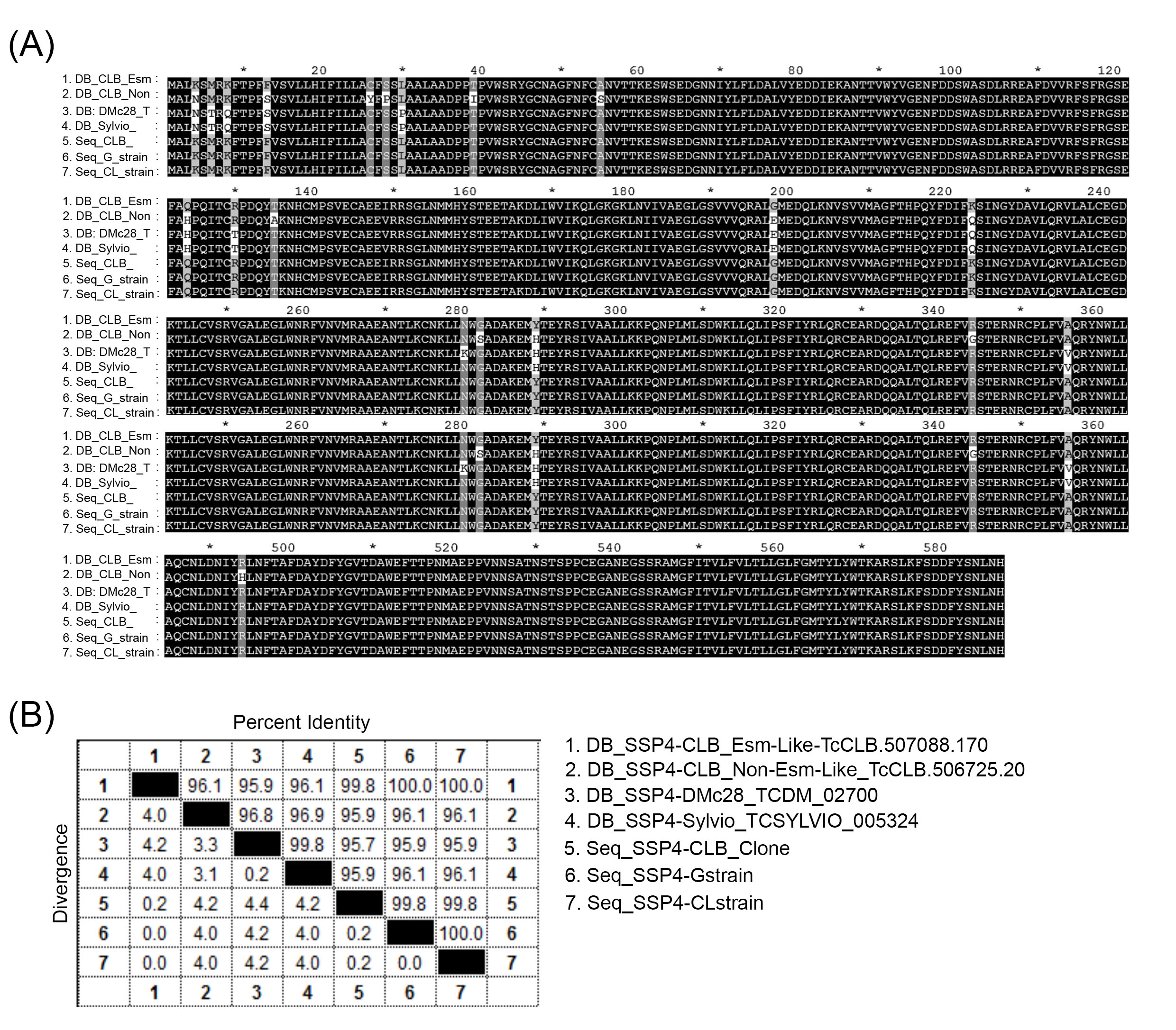

Supplement: FIGURE S2 — Ssp-4 protein alignment revealed a conserved backbone between different T. cruzi strains. (A) Sequences were aligned in the BioEdit program using the ClustalW algorithm. Regions with 100% identity between the sequences are shown in black. White and gray regions represent amino acid divergence in at least one of the aligned sequences. In the legend, 1 and 2 represent the Esmeraldo-like haplotype and non-Esmeraldo-like haplotypes from the CL Brener database, respectively; 3 and 4 represent Dm28c and Sylvio database sequences, respectively; 5, 6 and 7 represent Ssp-4 coding sequences from the CL Brener clone and the G and CL strains isolated in this study, respectively. (B) Protein identity and divergence scores between all strains aligned. [file Image_2.JPEG]

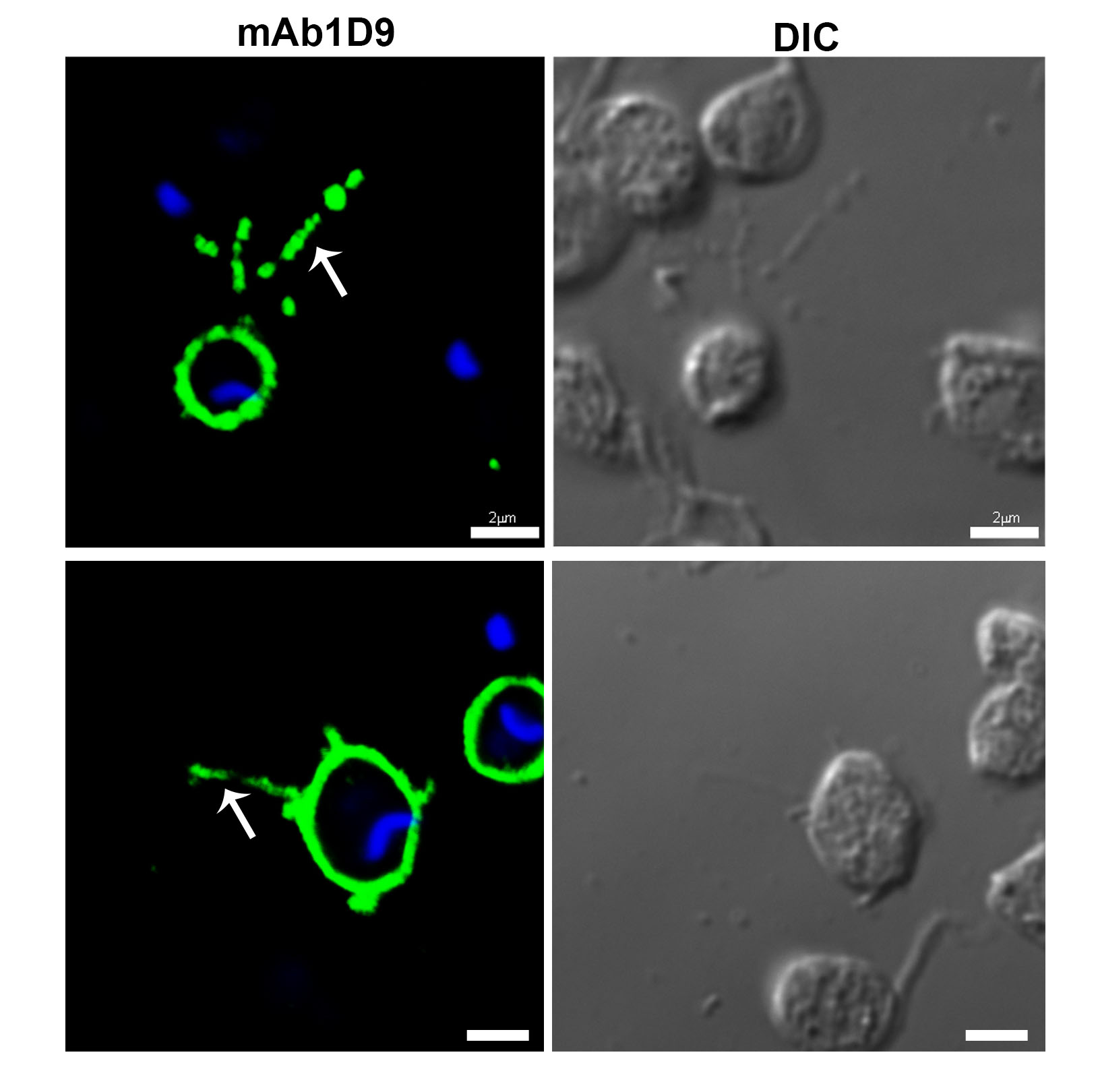

Supplement: FIGURE S3 — Vesicle trails covered with Ssp-4 carbohydrate epitopes on EAs of the G strain adhered to poly-L-lysine. Extracellular amastigotes (EAs) were attached onto coverslips coated with poly-L-lysine for 50 min at 37°C. Then, the parasites were fixed with 4% paraformaldehyde and incubated with blocking solution for 1 h. Samples were incubated with mAb1D9 (green) and DAPI (blue). Left panels: immunofluorescence images obtained from one plane. Arrows indicate released vesicle trails from parasites. Right panels: Differential interference contrast (DIC). Scale bar: 2 μm. [file Image_3.JPEG]
